# Comparative Genomic and Transcriptomic Analyses of *Mycobacterium kansasii* Subtypes Provide New Insights Into Their Pathogenicity and Taxonomy

**DOI:** 10.3389/fcimb.2020.00122

**Published:** 2020-03-24

**Authors:** Qingtian Guan, Roy Ummels, Fathia Ben-Rached, Yara Alzahid, Mohammad S. Amini, Sabir A. Adroub, Jakko van Ingen, Wilbert Bitter, Abdallah M. Abdallah, Arnab Pain

**Affiliations:** ^1^Pathogen Genomics Laboratory, BESE Division, King Abdullah University of Science and Technology (KAUST), Thuwal, Saudi Arabia; ^2^Department of Medical Microbiology and Infection Control, Amsterdam University Medical Centers, Amsterdam, Netherlands; ^3^Department of Medical Microbiology, Radboud UMC Center for Infectious Diseases, Radboud University Medical Center, Nijmegen, Netherlands; ^4^Department of Basic Medical Sciences, College of Medicine, QU Health, Qatar University, Doha, Qatar; ^5^Center for Zoonosis Control, Global Institution for Collaborative Research and Education (GI-CoRE), Hokkaido University, Sapporo, Japan

**Keywords:** non-tuberculous mycobacteria, comparative genomics, *M. kansasii* subtypes, *espACD* operon, virulence factor

## Abstract

*Mycobacterium kansasii* is an important opportunistic pathogen of humans and has a close phylogenetic relationship with *Mycobacterium tuberculosis*. Seven subtypes (I–VII) have been identified using molecular biology approaches, of which subtype I is the most frequent causative agent of human disease. To investigate the genotypes and pathogenic components of *M. kansasii*, we sequenced and compared the complete base-perfect genomes of different *M. kansasii* subtypes. Our findings support the proposition that *M. kansasii* “subtypes” I-VI, whose assemblies are currently available, should be considered as different species. Furthermore, we identified the exclusive presence of the *espACD* operon in *M. kansasii* subtype I, and we confirmed its role in the pathogenicity of *M. kansasii* in a cell infection model. The *espACD* operon is exclusively present in mycobacterial species that induce phagosomal rupture in host phagocytes and is known to be a major determinant of ESX1-mediated virulence in pathogenic mycobacteria. Comparative transcriptome analysis of the *M. kansasii* I-V strains identified genes potentially associated with virulence. Using a comparative genomics approach, we designed primers for PCR genotyping of *M. kansasii* subtypes I-V and tested their efficacy using clinically relevant strains of *M. kansasii*.

## Introduction

Nontuberculous mycobacteria (NTM) are increasingly being recognized as important opportunistic pathogens of humans. Reports have shown that more than 50 species of mycobacteria are associated with human diseases (Griffith et al., 2007), and *Mycobacterium kansasii* is the one of the most common causes of NTM disease in South America, South Africa and Europe (Hoefsloot et al., 2013).

*M. kansasii* was named after it was isolated from two patients suspected of having tuberculosis in Kansas, USA; it was previously known as the “yellow bacillus” (Pollak, 1953). This raised attention because it is difficult to treat patients co-infected with human immunodeficiency virus (HIV) (Levine and Chaisson, 1991; Corbett et al., 1999). In addition to systemic infection, *M. kansasii* also causes lung, cervical lymph node, and skin infections (Breathnach et al., 1995). Seven major subtypes have been described based on PCR-restriction fragment length polymorphism (RFLP) of *hsp65* and internal transcribed spacer (ITS) (Taillard et al., 2003), while subtype I remains the most commonly isolated from clinical environments (Taillard et al., 2003; Borówka et al., 2017; Machado et al., 2018). A recent study proposed that the “subspecies” of *M. kansasii* should be considered as new species (Tagini et al., 2019).

Previous phylogenetic studies have suggested that *Mycobacterium marinum* (Stinear et al., 2008), *Mycobacterium lacus* (Tortoli et al., 2017), *Mycobacterium decipiens* (Brown-Elliott et al., 2018), *Mycobacterium shinjukuense* (Saito et al., 2011), *Mycobacterium. riyadhense* (Fedrizzi et al., 2017; Guan et al., 2019; Sapriel and Brosch, 2019) along with *M. kansasii* are closely related to the free-living ancestor of the *Mycobacterium tuberculosis* complex (MTBC). Phylogenetically, *M. kansasii* is closely related to *M. tuberculosis* (Wang et al., 2015), and the clinical manifestation of *M. kansasii* infections also shows significant overlap with the clinical profile of *M. tuberculosis* infections. *M. kansasii* has been suggested to represent the environmental ancestor of *M. tuberculosis* and can serve as a potential model organism to study evolutionary aspects of the switch from an opportunistic environmental pathogen to a professional host-restricted pathogen (Wang et al., 2015).

The pathogenesis of mycobacteria depends on the secretion of key virulence factors by the ESX-1 secretion system, which is absent in the vaccine strain *Mycobacterium bovis* BCG (Mahairas et al., 1996; Pym et al., 2002; Hsu et al., 2003). Substrates of this secretion system are responsible for phagosomal escape of pathogenic mycobacteria in macrophages and thereby for successful completion of the intracellular infection cycle (van der Wel et al., 2007). The *espACD* operon (*Rv3616c*~*Rv3614c, espA, espC*, and *espD*), a group of non-RD1 loci (Fortune et al., 2005; MacGurn et al., 2005), is essential for ESX-1 function, and its presence in the NTM pathogen *M. kansasii* subtype I is of particular interest in this study.

To explore the complexity of *M. kansasii* subtypes and understand why *M. kansasii* subtype I is most commonly associated with human diseases, we generated high-quality genomes of all five available subtypes of *M. kansasii* using a combination of Illumina and Pacific Biosciences sequencing technologies. Furthermore, we undertook a comprehensive comparative genomics and transcriptomics approach to identify components of potential *M. kansasii* subtype I virulence factors, as well as establish the role of the *espACD* operon in the pathogenicity of *M. kansasii*. By utilizing genome sequences, we also designed PCR-based genotyping primers for distinguishing *M. kansasii* subtypes.

## Methods and Materials

### Ethics Statement

The research protocol was approved by the Institutional Biosafety and Bioethics Committee of King Abdullah University of Science and Technology (Jeddah, Saudi Arabia; #18IBEC23). We confirm that all adult subjects provided informed consent, and a parent of the child participant provided informed consent on his behalf. Written informed consent was given.

### Bacterial Strains and Cell Culture Conditions

We collected five environmental *M. kansasii* strains, designated KAUST-I to KAUST-V, that were originally isolated from either water or soil samples from five different European countries. Sixteen gDNA samples isolated from clinically relevant strains were also included in the study. These strains were collected from Radboud UMC Center of Infectious Diseases in the Netherlands. The isolates we collected were subtyped using the *hsp65* gene as discussed before (Telenti et al., 1993) and subtype I-V were found, which are mostly isolated in many clinical cases (Taillard et al., 2003; Houben et al., 2012; Borówka et al., 2017; Machado et al., 2018) and no subtype VI and VII was detected.

*M. kansasii* subtypes I-V (strains KAUST-I to KAUST-V) were grown in Middlebrook 7H9 (Saitoh et al., 2000) broth with 0.5% glycerol, 0.05% polysorbate 80 and OADC (0.85% sodium chloride, 5% bovine albumin, 2% dextrose, 0.003% catalase) at 37°C. The human monocytic cell line THP-1 (ATCC® TIB-202™) was grown in Roswell Park Memorial Institute medium (RPMI)-1640 supplemented with 10% fetal bovine serum, 100 μg/mL penicillin and 100 μg/mL streptomycin at 37Â°C with 5% CO_2_.

### DNA Isolation, Library Preparation, Assembly, and Genome Annotation

*M. kansasii* subtypes I-V DNA were isolated using a phenol-chloroform extraction protocol (Belisle and Sonnenberg, 1998). The extracted genomic DNA molecules were then sequenced using PacBio RSII sequencer with a 10 kb SMRT library. In parallel, genomic DNAs from *M. kansasii* subtypes I-V were sheared into ~500 bp fragments using Covaris^TM^. Paired-end, Illumina TruSeq PCR-Free^TM^ libraries were generated following the manufacturer's instruction, and the libraries were sequenced on a HiSeq2000 platform. The genomes were assembled into contigs using Canu (Koren et al., 2017) and corrected using Pilon (Walker et al., 2014) with PCR-free Illumina reads, and the complete assemblies were annotated by Prokka (Seemann, 2014).

### Genotyping and Comparative Genomics of *M. kansasii*

To gain a better understanding of the *M. kansasii* pan-genome and develop a quick and accurate genotyping protocol, we downloaded forty *M. kansasii* genome sequences available on the NCBI genome database before Oct. 15th, 2018. We compared the phylogeny of these strains based on the *hsp65* gene and ITS using the RaxML maximum-likelihood method (Stamatakis, 2014) with 100,000 replicates. Given that the standard method for reconstructing phylogenies of closely related microbes is the core-genome single-nucleotide polymorphism sites (SNPs) typing method, we also called the SNPs from the downloaded strain assemblies using Parsnp from the Harvest suite (Treangen et al., 2014) and generated the phylogenomic relationship map of *M. kansasii*. The phylogenetic tree was generated based on 135,969 core SNPs from each of the assemblies using the RaxML maximum-likelihood method (Stamatakis, 2014) with 100,000 replicates. In addition, the average nucleotide identity (ANI) between every two assemblies was calculated by OrthoANI (Lee et al., 2016). To determine the paralogous gene groups of the *M. kansasii* subspecies, the predicted protein sequences of *M. kansasii* subtypes I–V and *M. tuberculosis* were analyzed using OrthoMCL (Li et al., 2003) with a 50% identity cut-off and an inflation parameter of 1.5. The ortholog groups were visualized as a Venn diagram with the R VennDiagram package (Chen and Boutros, 2011). Mauve (Darling et al., 2004) was used to align the genomes of the subtypes to reveal the structure of the backbones of the chromosomes. The whole-genome comparison against subtype I was analyzed and visualized with BLAST Ring Image Generator (BRIG) (Alikhan et al., 2011). The uniqueness of singletons from each subtype and unique ortholog groups was further examined by BlastN (Version 2.2.26) against each other with 60% identity and an E-value of 0.00001 as the cutoff. The functional annotation of these genes was analyzed by EggNOG (Huerta-Cepas et al., 2016).

### Investigation of the Role of the *espACD* Operon in *M. kansasii* Pathogenicity

During our comparative genome analysis of the *M. kansasii* subtypes and the list of genes that are uniquely present in the subtype I, we have observed the presence of an *espACD* operon, which is known to have an important role in ESX-1 secretion (Ates and Brosch, 2017). To investigate the functional role of the *espACD* operon in *M. kansasii* subtype I, we constructed the pSMT3-*espACD*-GFP plasmid, and it was transformed into *M. kansasii* subtype II using the electroporation method (Goude et al., 2015) to investigate the function of the *espACD* operon.

The *espACD* operon of *M. kansasii* subtype I was amplified with the primers EspACD-HindIII (TTTTAAGCTTCGGGACTTGCGCTTAGTCTG) and EspACD- AflII (TTTTCTTAAGGTGGCCGCCCGTTTATGTAG). The DNA fragments were digested with HindIII and AflII and cloned into an AflII-restriction site containing a variant of pSMT3 digested with the same enzymes. The resulting plasmid, pSMT3-*espACD-*GFP, is a shuttle plasmid that is difficult to incorporate directly into *M. kansasii*. Therefore, we introduced the OriT region of pRAW (Ummels et al., 2014) and introduced the plasmid into *Mycobacterium marinum*. Subsequently, we introduced the plasmid pSMT3-*espACD*-GFP into *M. kansasii* through conjugation.

Cultures of the different subtypes of *M. kansasii* (wild-type subtype I, wild-type subtype II, subtype II transferred with pSMT3-*espACD-*GFP, and subtype II transferred with pSMT3-GFP) with an OD_600nm_ = 0.8–1.0 were prepared. Simultaneously, THP-1 cells (Tsuchiya et al., 1980) were counted using a haemocytometer and diluted to seed half a million cells per well in 24-well plates in the presence of 25 ng/mL phorbol myristate acetate (PMA) to allow the cells to differentiate into macrophages and adhere overnight at 37°C with 5% CO_2_. After their differentiation, the complete medium was replaced with RPMI+10% FCS without antibiotics and incubated for 3 h. The macrophages were incubated in triplicate with *M. kansasii* KAUST-I, KAUST-II, KAUST-II-pSMT3-*espACD*-GFP, and KAUST-II-pSMT3-GFP, with a multiplicity of infection (MOI) = 5, or incubated with only medium. After 2 h of infection, the macrophages were washed with PBS and incubated with fresh medium without antibiotics. The cells were then treated with 1 mL of 1% Triton X-100 for 10 min at 37°C and collected at different time points after infection (0, 24, 48, and 72 h). Then, the obtained lysates were diluted to 1:10, 1:100, and 1:1,000, and 100 μL of each dilution was subsequently spread on Middlebrook 7H10 agar plates. The survival rate was evaluated as the percentage of colony forming units (CFUs) at different time points, taking the number of CFUs at time point “0” as the reference.

### Development of Genotyping Primers for *M. kansasii* Subtypes

The unique genes within each singleton pool were further investigated. We used BlastN against the non-redundant (nr) nucleotide database for the unique genes and refined the list of selected unique genes after comparison against the other species in the nr database. The unique genes were also confirmed by the binary alignment map (BAM) files generated by cross-mapping of Illumina reads to each subtype. The primer sets that target the unique genes are presented in Table 1. Primer sets that target the 16S rRNA V2~V4 regions were used as internal controls. GoTaq^TM^ Green Master Mix was used for the amplification, and the cycling conditions for these primers consisted of preheating at 95°C for 2 min, followed by 30 cycles of denaturation at 95°C for 30 s, annealing at 62°C for 30 s and extension at 72°C for 30 s. The final extension was performed at 72°C for 10 min after 30 cycles.

**Table 1:** Primer sets designed from selected sequences of *M. kansasii* subtypes I-V.

**Primer ID**	**Target gene**	**Amplicon Length (bp)**	**Forward Primer (5**′**-3**′**)**	**Reverse Primer (5**′**-3**′**)**
DPT1^*a*^	*kaust1_03087*	199	GTTCGTCTCGATTTCGCAGC	GAATCACGCGCCTTGATGAC
DPT2	*kaust2_05863*	312	TTTCGGACAATGACGGCGGACG	ATGCAGTGTCCGGCAAAGGGGT
DPT3	*kaust3_04009*	250	TGGCGGGTGTGTTGATGATGGC	ATCAGCGGCAACGGCGGTAA
DPT4	*kaust4_00078*	111	GATGGTCAAATCGAGCGACGAGGCG	GCGACGGGTTCATCGGCAGTGATT
DPT5	*kaust5_02691*	445	ACGCCTTGGAACGTGACCGTGA	ACTGATTCGTGGCCCGGATGGA
Internal control *kaust1_00615, kaust2_00230, kaust3_03982, kaust4_05573, kaust5_02753*^*b*^		591	TGGCGCATGCCTTGTGGTGGAA	TCCTGTTCGCTCCCCACGCTTT

a Diagnostic primer for subtype I.

b *Internal control that targets the 16S rRNA gene*.

### Transcriptome Analysis of *M. kansasii* Subspecies

Total RNA was extracted from 40 ml of exponential growth phase culture bacterial culture using the TRIzol protocol (Rio et al., 2010). Briefly, the bacterial cultures were centrifuged at 1,500 rcf for 15 min, suspended in 1 mL of TRIzol and incubated for 5 min. Then, 500 μL of zirconia beads (Biospec^TM^) were added and treated six times by beating at maximum speed for 30 s. Then, the mixture was centrifuged, and the upper layer was incubated with 200 μL of chloroform. After centrifugation at 4°C at maximum speed for 20 min, an equal volume of isopropanol was added to the aqueous layer. The mixture was centrifuged at 4°C at full speed, and the supernatant was discarded. Then, 1.5 mL of 70% cold ethanol was added and centrifuged for 10 min. The ethanol was discarded, and the RNA was air-dried. The RNA was suspended in 30 μl of RNase-free water and incubated at 60°C until all of the RNA was dissolved. The RNA was then stored at −80°C before library preparation. For library preparation, DNA was removed using Turbo^TM^ DNase, and rRNA was removed using the Invitrogen^TM^ Ribominus Kit. Strand-specific Illumina RNAseq libraries were prepared using the TruSeq kit following the manufacturer's manual, and the libraries were sequenced on a HiSeq2000 platform (Illumina^TM^). The RNAseq reads obtained were first trimmed using the Trimomatic program (Bolger et al., 2014) to remove adapters and low-quality reads (cutoff: Q30). The “clean reads” were further mapped to the annotated genomes of *M. kansasii* subtypes I-V. To compare the differences between *M. kansasii* subtype I and the four other subtypes, the transcriptomes of the 3,761 one-to-one orthologs obtained from OrthoMCL were compared. The RNAseq reads were mapped to each genome with HISAT2 (Pertea et al., 2016), and the reads mapped to each gene were counted by HTSeq (Anders et al., 2015) with union mode. DESeq2 (Anders and Huber, 2013) was used to call the differentially expressed genes. Genes with a padj value < 0.01 and an absolute Log_2_ fold change value greater than two were selected and further analyzed. The same method was applied to determine the differentially expressed genes (DEGs) in the other four *M. kansasii* subtypes.

## Results

### Comparative Genomics of *M. kansasii* Subtypes I–V

**Figure 1:**
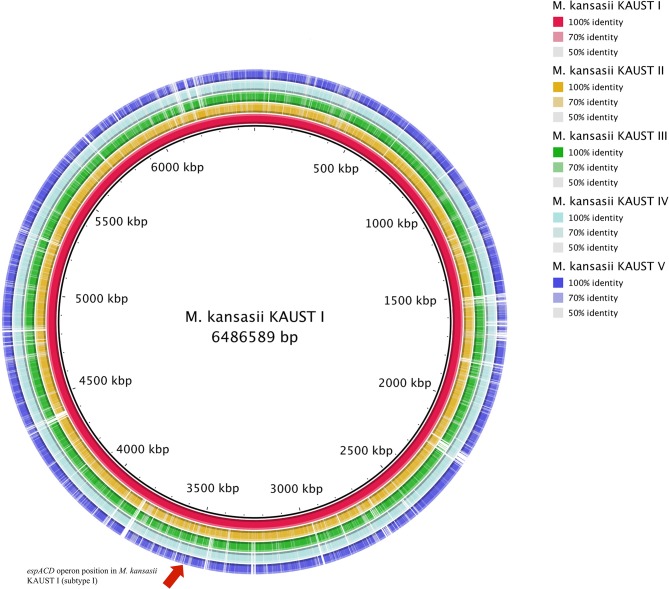
Circular map representation of the *M. kansasii* subtype II–subtype V genome showing BlastN similarities to *M. kansasii* subtype I. Each ring of the circle corresponds to a specific complete genome as referred to in the figure keys on the right. The *espACD* operon position in *M. kansasii* KAUST I is indicated by an arrow.

To study the variation in the *M. kansasii* subtypes, we obtained isolates belonging to each subtype and used a combination of Illumina and SMRT (PacBio) sequencing methods to assemble genomes into single base-perfect contigs. All of the bacterial chromosomes of *M. kansasii* subtypes I-V were assembled into a single contig each, and the genome of *M. kansasii* subtype III included a large new 301,558 bp circular plasmid pMKIII01 (Supplementary Figure 1) that has not been described before. The sequencing depths of each of the assemblies (subtype I–V) from PacBio were 251X, 122X, 98X, 148X, and 158X, respectively. The comparison of these genome assemblies with *M. tuberculosis* H37Rv, *M. marinum* M and the type strain *M. kansasii* 12478 are shown in Table 2. The comparison of our assemblies with previously published genomes from the same strains is described in Supplementary Table 1. The backbone structure of subtypes I–V is shown in Supplementary Figure 2, and the BlastN comparison of subtypes II–V to subtype I is shown in Figure 1.

**Table 2:** Comparison of general features/gene families of *M. kansasii* subtypes I-V with those of other Mycobacteria.

**Features**	***M. kansasii* ATCC12478**	***M. kansasii* KAUST-I**	***M. kansasii* (*Mycobacterium pseudokansasii*) KAUST-II**	***M. kansasii* (*Mycobacterium parakansasii*) KAUST-III**	***M. kansasii* (*Mycobacterium probekansasii*) KAUST-IV**	***M. kansasii* (*Mycobacterium novokansasii*) KAUST-V**	***M. tuberculosis* H37Rv**	***M. marinum* E11**
Genome Size (M)	Chromosome:6.43 Plasmid pMK12478:0.14	6.52	6.40	Chromosome:6.39 pMKIII01: 0.30	6.24	6.09	4.41	Chromosome:6.34 pRAW:0.11
GC content (%)	Chromosome:66.2 Plasmid pMK12478: 65.8	66.20	66.13	Chromosome:66.22 pMKIII01:64.48	66.20	66.36	65.6	Chromosome:65.8 pRAW: 64.1
CDS	Chromosome: 5,631 Plasmid pMK12478:152	5209	5211	Chromosome:5223 pMKIII:344	5145	5009	3,906	Chromosome: 5,149 pRAW:94
tRNA	Chromosome: 46 Plasmid: NA	55	56	Chromosome:52 pMKIII01:NA	50	54	45	Chromosome: 47 pRAW:NA
rRNA	Chromosome: 3 Plasmid: NA	3	3	Chromosome:3 pMKIII01:NA	3	3	3	Chromosome: 3 pRAW: NA
Reference	Cole et al., 1998	This study	This study	This study	This study	This study	Wang et al., 2015	Weerdenburg et al., 2015

**Figure 2:**
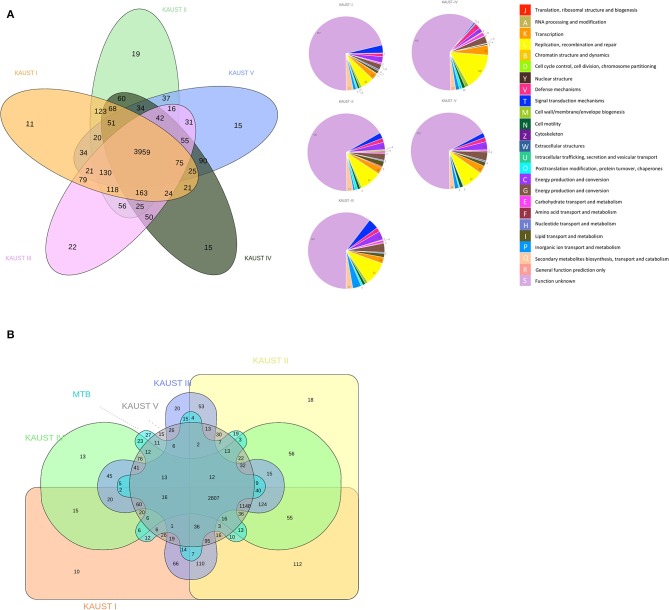
Comparative genomics study of the chromosomes of *M. kansasii* subtypes I to V. Venn Diagram of the ortholog groups and paralogue groups shared by **(A)** the five *M. kansasii* subtypes and **(B)** the five subtypes with *M. tuberculosis* H37Rv. The number represents the ortholog groups or paralogue groups present in the particular types.

The ortholog groups cluster across the genomes of the different *M. kansasii* subtypes, and the functional annotation categories of the paralogues/singletons are presented in Figure 2A. The number of unique singletons of each subtype that did not appear in the Venn diagram from *M. kansasii* subtypes I-V was 555, 628, 608, 756, and 642, respectively, most of which were proteins with unknown functions. This means that all subtypes have a similar number of singletons, indicating that they have similar genetic headroom and evolutionary pressures. The comparison of *M. kansasii* subtypes and *M. tuberculosis* H37Rv is shown in Figure 2B. A detailed analysis of the genes shared between *M. kansasii* subtype I and *M. tuberculosis* H37Rv is presented in Supplementary Table 2.

One of the striking findings in the comparative genomic analysis was that one copy of the *espACD* operon (*kaust1_3087*~*kaust1_3085*) is present exclusively in subtype I (Figure 1, Supplementary Figure 3). We examined whether these *espACD* loci are involved in the virulence of *M. kansasii*.

### Genotyping of *M. kansasii*

**Figure 3:**
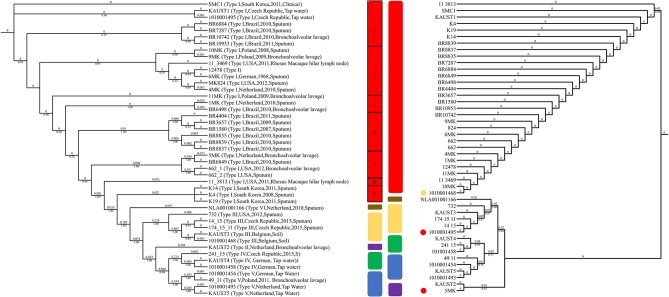
Genotyping of *M. kansasii* using ITS and SNPs from the complete genome assemblies. The maximum-likelihood tree based on the internal transcribed spacer (ITS) sequences of 45 *M. kansasii* strains is shown on the right. The maximum-likelihood phylogenetic tree based on 135,969 SNPs from the core genome of *M. kansasii* subtypes is shown on the left. Bootstrap support values are indicated on the branch as a percentage of 100,000 replicates. The branch length is ignored for illustration purposes and displayed on each branch. The color code for each genotype: red: *M. kansasii* subtype I; purple: *M. kansasii* subtype II; yellow: *M. kansasii* subtype III; green: *M. kansasii* subtype IV; blue: *M. kansasii* subtype V; brown: *M. kansasii* subtype VI. The strains within *M. kansasii* subtype I are separated with black rectangles in the figure. The circles indicate the difference between the ITS and SNP genotyping method, and colors represent the genotypes suggested by ANI clustering.

Various molecular methods for genotyping *M. kansasii* have been developed for comparison and strain identification (Zhang et al., 2004). To study the subtypes in detail, we analyzed the available 45 sequenced *M. kansasii* genome datasets. The analysis revealed that the phylogenetic tree that was constructed based on the ITS sequences grouped the *M. kansasii* subtypes incorrectly in some cases (Figure 3) due to limited information provided in the sequences (Supplementary Figure 4). *In silico* genotyping of the subtypes of the 45 *M. kansasii* strains based on whole-genome core SNPs and ANI revealed that there are six major subtypes of *M. kansasii* that have been sequenced in full length so far (Figure 3, Supplementary Table 3). The ANI value between the subtypes of *M. kansasii* is high, which supports the idea that *M. kansasii* “subtypes” I-VI should be considered as different species; e.g., the ANI value between subtype VI and subtype III is 89.35–89.48, and the value between subtype II and subtype VI is 92.86–92.95. Therefore, we conclude that *M. kansasii* subtypes I-VI should be assigned as different species. This finding is in line with a recently published result describing the ANI values between *M. kansasii* subtypes (Tagini et al., 2019) in which the authors proposed that *M. kansasii* subtypes III, V and VI should be considered as independent species rather than subspecies.

### Comparative Transcriptome Analysis

**Figure 4:**
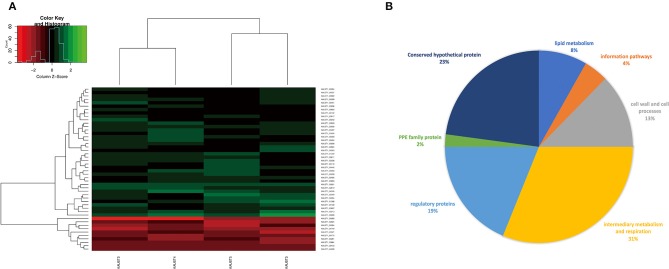
**(A)** Gene expression profiles (log_2_ fold change) of the differentially expressed genes of *M. kansasii* subtypes II-V in comparison to *M. kansasii* subtype I. The red color represents upregulated genes, and the green color represents downregulated genes compared with the control strain. padj < 0.01 and Log_2_FC > 2 were used as the cutoff for the selection of the genes. **(B)** Pie-chart showing a subdivision of all differentially expressed genes based on functional categories.

To reveal the differences in expression profiles of *M. kansasii* subtypes, we performed a global transcriptome analysis across *M. kansasii* subtypes I-V grown *in vitro*, focusing on the changes in gene expression profiles during the exponential growth of the bacilli in Middlebrook 7H9 medium. To investigate this, RNA was extracted from three independent exponential phase cultures of *M. kansasii* subtypes I-V. Data quality was assessed using Euclidean distance matrices and demonstrated high levels of reproducibility between the biological replicates (Supplementary Figure 5). After filtering, a total of 38 genes were uniquely downregulated and 10 genes were upregulated in *M. kansasii* subtype I in comparison with *M. kansasii* subtypes II-V within the one-to-one ortholog groups (Figure 4A) (Supplementary Table 4). Analysis of differential expression in *M. kansasii* subtype I identified changes in genes involved in a variety of cellular processes, mostly genes associated with metabolic, respiratory, regulatory and cell wall-associated processes (Figure 4B).

We noted that a substantial number of genes that are likely essential and required for mycobacterial growth (Sassetti et al., 2003; Weerdenburg et al., 2015) were differentially regulated in *M. kansasii* subtype I, including genes involved in cell wall-associated processes (*mpt63*) and lipid metabolism (*accD4, mbtC*) (Supplementary Table 4). In addition, many of these differentially regulated genes encode proteins of unknown function and hence need to be further characterized (Figure 4A). However, we did not notice any well-known virulence genes in the list.

To determine the functional classifications and pathways of the DEGs associated with each subtype, all upregulated DEGs from each subtype were analyzed with EggNOG Mapper (Huerta-Cepas et al., 2016) (Supplementary Table 5). The different KEGG pathways associated with the DEGs were as follows: three pathways for *M. kansasii* subtype I, twenty-one pathways for *M. kansasii* subtype II, nine pathways for *M. kansasii* subtype IV and 14 pathways for *M. kansasii* subtype V. Notably, metabolic pathways were significantly dominant in all subtypes.

### Complementation of the *espACD* Operon Recovers the Pathogenicity of the Non-pathogenic *M. kansasii* Subtype II

*M. kansasii* subtype I contains five ESX systems, and the overall arrangement of the five ESX systems is similar to that in *M. tuberculosis* H37Rv (Supplementary Figure 6). This includes the ESX-1 region, which is important for virulence in *M. tuberculosis* (Conrad et al., 2017; Tiwari et al., 2019). Previous research has shown that the ESX-1 substrate EsxA is secreted by both *M. kansasii* subtype I and subtype V (Houben et al., 2012). Surprisingly, the same study also showed using electron microscopy that only *M. kansasii* subtype I is able to escape from the phagolysosome (Houben et al., 2012).

**Figure 5:**
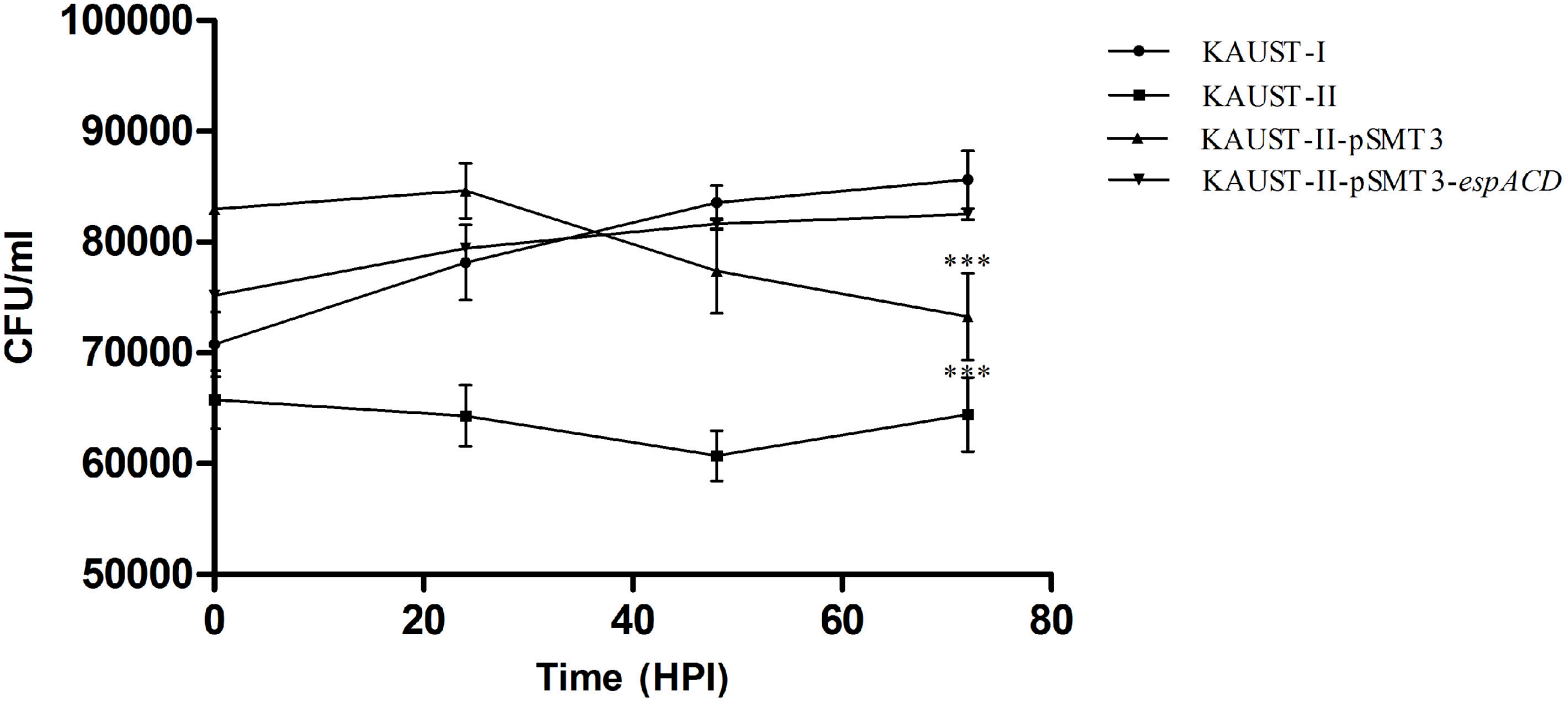
Complementation of the *espACD* operon in *M. kansasii* subtype II with that from *M. kansasii* subtype I is crucial for its virulence. The functional complementation experiment revealed that the *espACD* operon plays a significant role in *M. kansasii* subtype II pathogenicity, at least in THP-1 cells. Intracellular CFU count showing the bacterial load in THP-1 macrophages infected with KAUST-I (•), KAUST-II (•), KAUST-II-pSMT3 (▴) and KAUST-II-pSMT3-*espACD* (▾) at a MOI of 5. The infected macrophages were lysed at 0, 24, 48, and 72 h time-points post-infection and three dilutions of the released mycobacterial cells were plated on 7H10 agar plates. CFU were counted and recorded after 15 days of plating. Experiments were performed with three replicates, and Student's *t*-test for significance was calculated with the level of significance shown (***highly significant difference, *p* < 0.01).

To test whether this *espACD* locus could affect the virulence of *M. kansasii*, we cloned the *espACD* gene cluster of subtype I on a shuttle plasmid and introduced this plasmid into subtype II, which does not have the *espACD* operon. We first confirmed that there are no *in vitro* growth differences among the tested strains (Supplementary Figure 7). Subsequently, differentiated THP-1 monocytes were infected with these strains. After 3 days, we observed a significant decrease in the survival rate of wild-type *M. kansasii* subtype II and the same bacterial strain harboring the control plasmid pSMT3-GFP (Figure 5). On the other hand, wild-type subtype I and the subtype II strain transfected with pSMT3-*espACD*-GFP had significantly increased CFU numbers. We concluded that the presence of the *espACD* operon was able to increase the virulence of *M. kansasii* subtype II (Figure 5).

### *M. kansasii* Subspecies Genotyping

**Figure 6:**
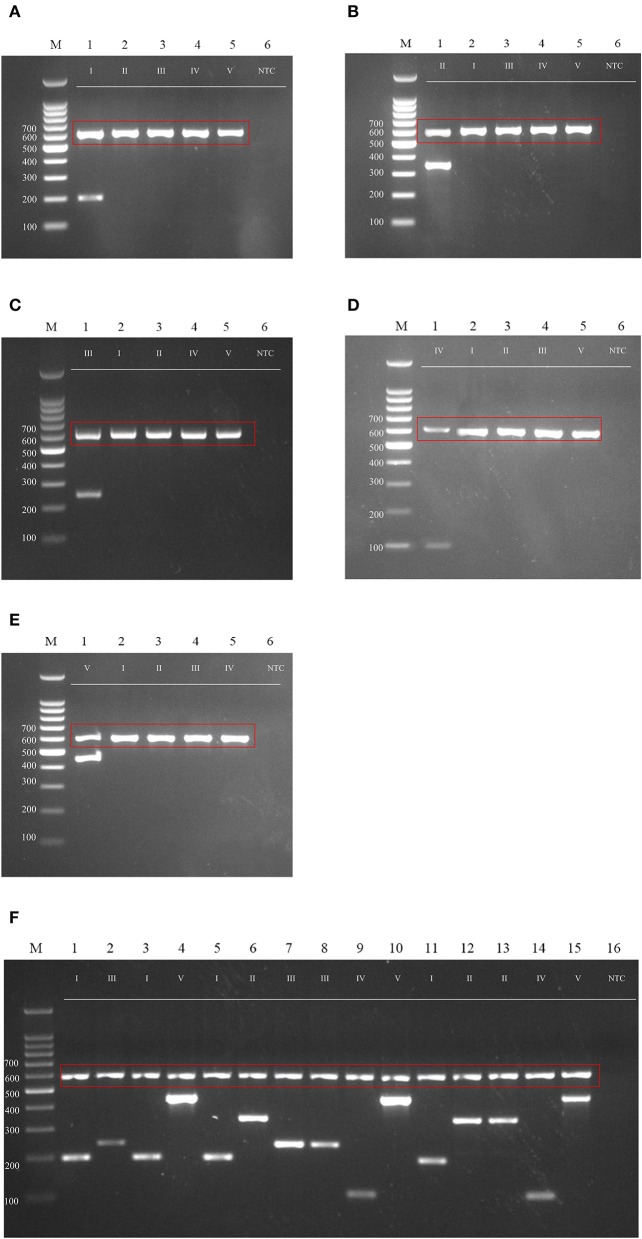
Agarose gel (2%) electrophoresis of diagnostic PCR tests for *M. kansasii* subtypes I-V. Lane M: DNA markers. The gDNA used for testing is labeled on the top, and the size of the ladder is labeled on the left side of each gel picture. The genotyping results for *M. kansasii* subtypes I-V are listed in **(A–E)**. The clinical samples tested with the cocktail of primers DP1-5 is shown in **(F)**. The 16S primer control is shown in the red box. NTC, Non-template control.

Many genotyping methods have been applied for the subtyping of *M. kansasii*. Several of those methods either failed to distinguish all of the subtypes, such as AccuProbe^TM^ (Tortoli et al., 1996) and INNO LiPA^TM^ (Suffys et al., 2001), or involved sequencing (Park et al., 2000) or restriction enzyme digestion (Bakuła et al., 2013) steps. To simplify the genotyping method for these different subtypes, especially the more virulent subtype I, we developed a genotyping method that can rapidly and accurately distinguish *M. kansasii* subtypes I-V by PCR (Figures 6A–E). The primer sets accurately identify *M. kansasii* subtypes and can serve as part of an accurate and rapid diagnostic protocol in a clinical setting (Figure 6F) to overcome misdiagnosis or extensive lab work using the *hsp65* and ITS regions (Figure 3).

## Discussion

We obtained high-quality genomes of the five most frequently observed types of *M. kansasii* by applying a combination of short reads and long reads from the Illumina and PacBio platforms, respectively. This allowed us to undertake a comprehensive genome comparison at single base-pair resolution and define the key hallmarks of the five subtypes of sequenced *M. kansasii* strains.

As expected, owing to the environmental niche of this bacteria, *M. kansasii* subtypes I-V share a large number of orthologs that are not present in *M. tuberculosis* H37Rv (1148). Toxin/antitoxin (T/A) systems were previously only reported in *M. tuberculosis* while recent studies shows the expansion of the T/A systems from NTMs to MTBC (Guan et al., 2019; Sapriel and Brosch, 2019). The comparison of the *M. kansasii* subtype I and *M. tuberculosis* H37Rv genomes reveal that they share 12 genes that are not present in other subspecies (Figure 2B, Supplementary Table 2). Two copies of the toxin and antitoxin proteins VapB12 and VapC4, which are known to be involved in the adaptation of *M. tuberculosis* (Sala et al., 2014), two mobile genetic elements genes within the RD3 of *M. tuberculosis* H37Rv (*rv1584c, rv1585c*) and one copy of a regulatory protein (*rv1129c*) that has been shown to be required for *M. tuberculosis* growth on cholesterol (Griffin et al., 2011) are uniquely present in *M. kansasii* subtype I and *M. tuberculosis* H37Rv. We also found two PPE family genes (*rv1801, rv1802*) that are present in subtype I, which was reported to be essential for endothelial-cell invasion and/or intracellular survival (Talaat et al., 2004; Jain et al., 2006). *M. kansasii* phylogenetically and clinically resembles *M. tuberculosis* (Wang et al., 2015), and the preservation of these genes during the reduction of the *Mycobacterium tuberculosis* complex (MTBC) genomes indicates that they may be important for virulence and pathogenicity.

pMKIII01 is a novel plasmid with a lower GC content (64.48%) than the *M. kansasii* subtype III chromosome, whose G+C content is 66.22%. This may suggest that this plasmid was not originally from *M. kansasii* subtype III and has been transformed into the bacterial cells during the evolutionary processes. The new plasmid pMKIII01 (Supplementary Figure 1) harbors a locus encoding a putative Type IV secretion system and a putative Type VII secretion system. This plasmid resembles the conjugative mycobacterial plasmids that have been discovered previously, such as the type-plasmid pRAW in *M. marinum* (Ummels et al., 2014), pMAH135 (Uchiya et al., 2015), and pMA100 (da Silva Rabello et al., 2012) of *Mycobacterium avium*, pMyong1 from *Mycobacterium yongonense* (Kim et al., 2013), pMK12478 (Veyrier et al., 2009) from *M. kansasii* 12478 and several plasmids from *Mycobacterium chimera* (van Ingen et al., 2017). The presence of a pRAW-like plasmid in *M. kansasii* subtype III confirms that these plasmids are widespread in the *Mycobacterium* genus, which seems to be logical, as they were shown to be conjugative (Ummels et al., 2014). The variation in the sizes of pRAW-like plasmids is significant, with a value of 301,558 bp; the pMKIII01 plasmid is 2.5 times the size of pRAW. Thus far, there is no evidence that pRAW-like plasmids are directly involved in virulence, but they have been instrumental in the evolution and duplication of the ESX systems (Dumas et al., 2016; Newton-Foot et al., 2016).

The commonly used method for diagnosis, which is based on the variation of *hsp65* and ITS sequences, either requires restriction enzyme digestion steps or cannot distinguish the *M. kansasii* subtypes accurately (Figure 3, Supplementary Figure 4). While the chromosomes of all five subtypes (I-V) are syntenic and contain primarily shared orthologs (Figure 1, Supplementary Figure 2) with a limited number of uniquely shared patterns of genes across all of them, the phylogenomic analyses (Figure 3) and the ANI figures revealed unusually high levels of nucleotide diversity amongst the *M. kansasii* complex (Supplementary Table 3). The complexity of *M. kansasii* is greater than we previously thought, and *M. kansasii* is too diverse to be considered as a single “species” (Figure 3) given that approximately >95–96% ANI values are considered to set the species boundary (Richter and Rosselló-Móra, 2009). This proposition is also consistent with the differences in virulence characteristics amongst the subtypes.

Hence, we propose to consider *M. kansasii* “subspecies” as six different species: *Mycobacterium pseudokansasii* (previously classified as *Mycobacterium kansasii* subtype II); *Mycobacterium parakansasii* (previously classified as *Mycobacterium kansasii* subtype III); *Mycobacterium probekansasii* (previously classified as *Mycobacterium kansasii* subtype IV); *Mycobacterium novokansasii* (previously classified as *Mycobacterium kansasii* subtype V); and *Mycobacterium eurokansasii* (previously classified as *Mycobacterium kansasii* subtype VI).

In line with this, the function of *M. kansasii* subtype I DEGs is diverse and includes aminopeptidase, ADP-ribose pyrophosphatase and phenyloxazoline synthase (Figure 4A, Supplementary Table 4), suggesting the diverse metabolic capacities of *M. kansasii* subtypes I and II–V.

There are now approximately one dozen ESX-1 substrates identified (Champion et al., 2014; Phan et al., 2018; Sala et al., 2018; Abdallah et al., 2019) in *M. tuberculosis*, and most of the genes coding for these ESX-1 substrates are located within the ESX-1 locus. Although ESX-1-secreted substrates are essential for virulence, their separate roles are not well defined. This is because the secretion of some of these substrates, such as EspA and EsxA (ESAT-6), is mutually dependent (Fortune et al., 2005; Houben et al., 2012). The genes located within the *espACD* operon (*rv3614*~*rv3616c*) are outside the ESX-1 loci. They are homologous to *rv3864*~*rv3867*, located within the ESX-1 locus and required for ESX-1 secretion for virulence (MacGurn et al., 2005). While no evidences that suggest there are any connections of these two groups of genes. Comparative genome analyses suggest that the *espACD* serves as a pathogenetic island (Simeone et al., 2012; Majlessi et al., 2015) which might be introduced independently by horizontal transfer from different species (Ates and Brosch, 2017). EspC associates with EspA in the cytoplasm and membrane, then polymerizes during secretion from *M. tuberculosisis*. EspC forms a filamentous structure in the cell envelope of *M. tuberculosis* hence affects ESX-1 secretion (Lou et al., 2017). We have confirmed that the *espACD* operon plays a crucial role in the pathogenicity of *M. kansasii*. Functional complementation of the *espACD* operon in wild-type *M. kansasii* subtype II was able to recover the persistence phenotype (Figure 5). It is probable that this increased persistence is induced by complementation of the ESX secretion system, which might be essential for bacteria survival within macrophages. Not surprisingly, *M. kansasii* subtype II-pSMT3-*espACD*-GFP was still less virulent than the wild-type *M. kansasii* subtype I because the *espACD* operon is under the control of EspR (Raghavan et al., 2008; Kumar et al., 2016) and PhoPR (Frigui et al., 2008), which are missing in the plasmid.

In conclusion, our study reveals that *M. kansasii* subtype I has several unique features in comparison to the established subtypes II-V, consistent with its pathogenicity characteristics. We also provide evidence that the *espACD* operon plays an important role in acquiring virulence, at least in *M. kansasii* subtype II. Our results support the notion that there are six subtypes of *M. kansasii*, and the high levels of nucleotide variation amongst the subtypes prompt us to propose that we should consider *M. kansasii* subtypes I–VI as different species. Finally, we developed a simple approach to distinguish *M. kansasii* subtypes I-V that can be applied in clinical settings.

## Data Availability Statement

The *M. kansasii* dataset is available at the European Nucleotide Archive (ENA) under study accession No. PRJEB32175.

## Ethics Statement

The studies involving human participants were reviewed and approved by The research protocol was approved by the Institutional Biosafety and Bioethics Committee of King Abdullah University of Science and Technology (Jeddah, Saudi Arabia; #18IBEC23). The patients/participants provided their written informed consent to participate in this study.

## Author Contributions

AP and AA conceived the study and obtained the funding and supervised the work. AP, QG, and AA designed the experiments. RU generated the strains for the complementation experiment. QG and FB-R performed the complementation experiment. YA, MA, and SA generated the Illumina data and helped in the analysis. QG generated the PacBio data, performed all the data analysis, and prepared the initial draft of the manuscript, followed by edits from AP, WB, AA, and JI. All authors have commented on various sections of the manuscript, which were finally curated and incorporated into the final version by QG, AP, and AA.

### Conflict of Interest

The authors declare that the research was conducted in the absence of any commercial or financial relationships that could be construed as a potential conflict of interest.
