# Supplementary material for: Comparative Genomic and Transcriptomic Analyses of Mycobacterium kansasii Subtypes Provide New Insights Into Their Pathogenicity and Taxonomy
Source: Front Cell Infect Microbiol. 2020 Mar 24;10:122. doi: 10.3389/fcimb.2020.00122 (PMC7105574; doi:10.3389/fcimb.2020.00122)
Supplement: Supplementary file 1 [file Data_Sheet_1.doc]

Supplementary Material

**Comparative genomic and transcriptomic analyses of *Mycobacterium kansasii* subtypes provide new insights into their pathogenicity and taxonomy**

Qingtian Guan^1^, Roy Ummels^2^, Fathia Ben-Rached^1^, Yara Alzahid^1^, Mohammad S. Amini^1^, Sabir A. Adroub^1^, Jakko van Ingen^3^, Wilbert Bitter^2^, Abdallah M. Abdallah^4,1#*^, Arnab Pain^1,5#*^

^1^Pathogen Genomics Laboratory, BESE Division, King Abdullah University of Science and Technology (KAUST), Thuwal-Jeddah, Kingdom of Saudi Arabia;

^2^Department of Medical Microbiology and Infection Control, Amsterdam University Medical Centers, Amsterdam, The Netherlands;

^3^Radboud UMC Center for Infectious Diseases, Department of Medical Microbiology, Radboud University Medical Center, Nijmegen, the Netherlands;

^4^Department of Basic Medical Sciences, College of Medicine, QU Health, Qatar University, Doha, Qatar

^5^Center for Zoonosis Control, Global Institution for Collaborative Research and Education (GI-CoRE); Hokkaido University, Sapporo, Japan

**#Contributed equally**

***Correspondence:**

**Abdallah M Abdallah (Abdallah.musa@qu.edu.qa)**

**Arnab Pain (arnab.pain@kaust.edu.sa)**

**
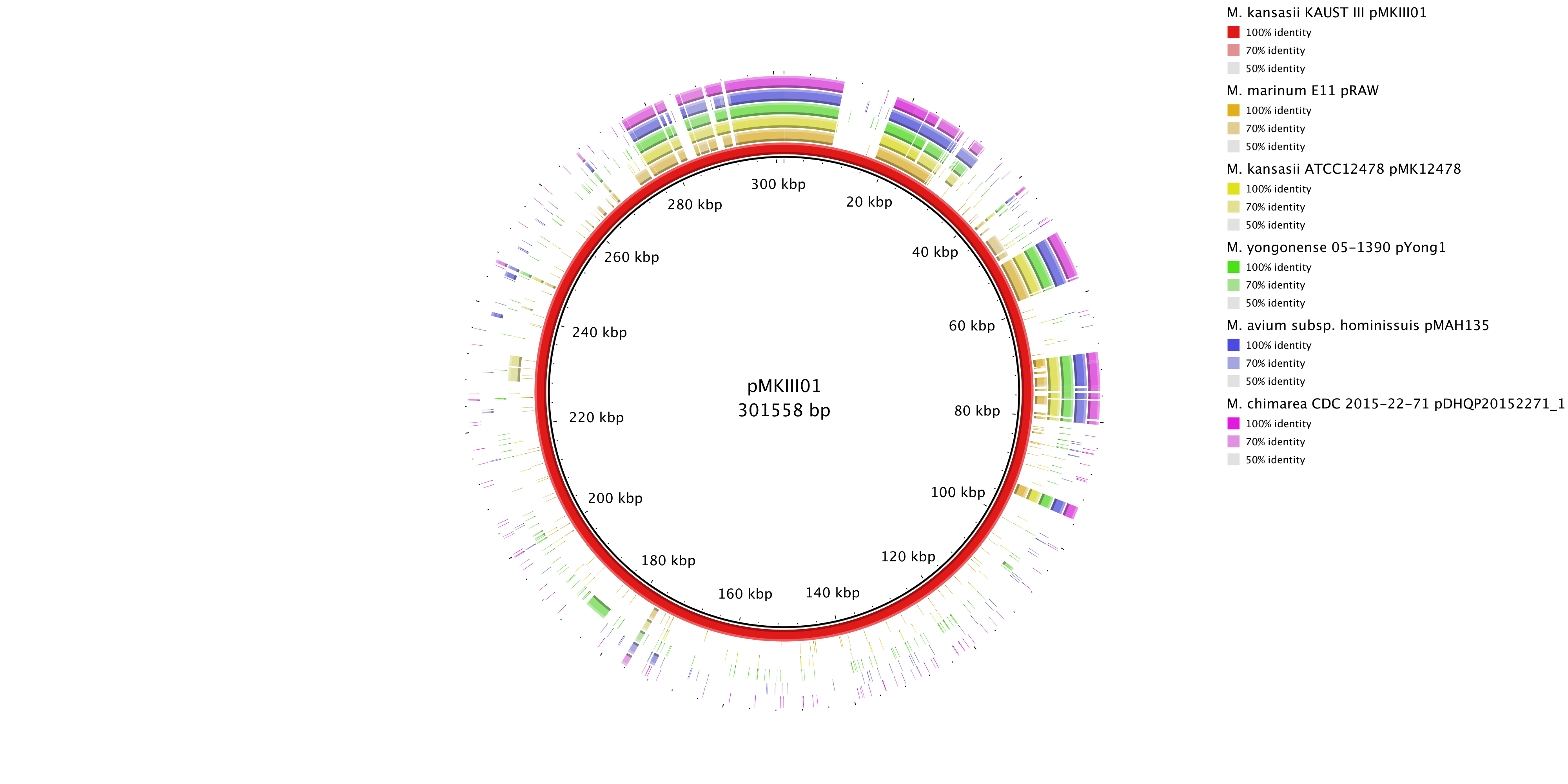
**

**Supplementary Figure 1.** Circular map of various Mycobacteria plasmids BlastN results using pMKIII01 as the reference in *M. kansasii* subtype III. The color code for each of the plasmids is listed in the right panel.

**
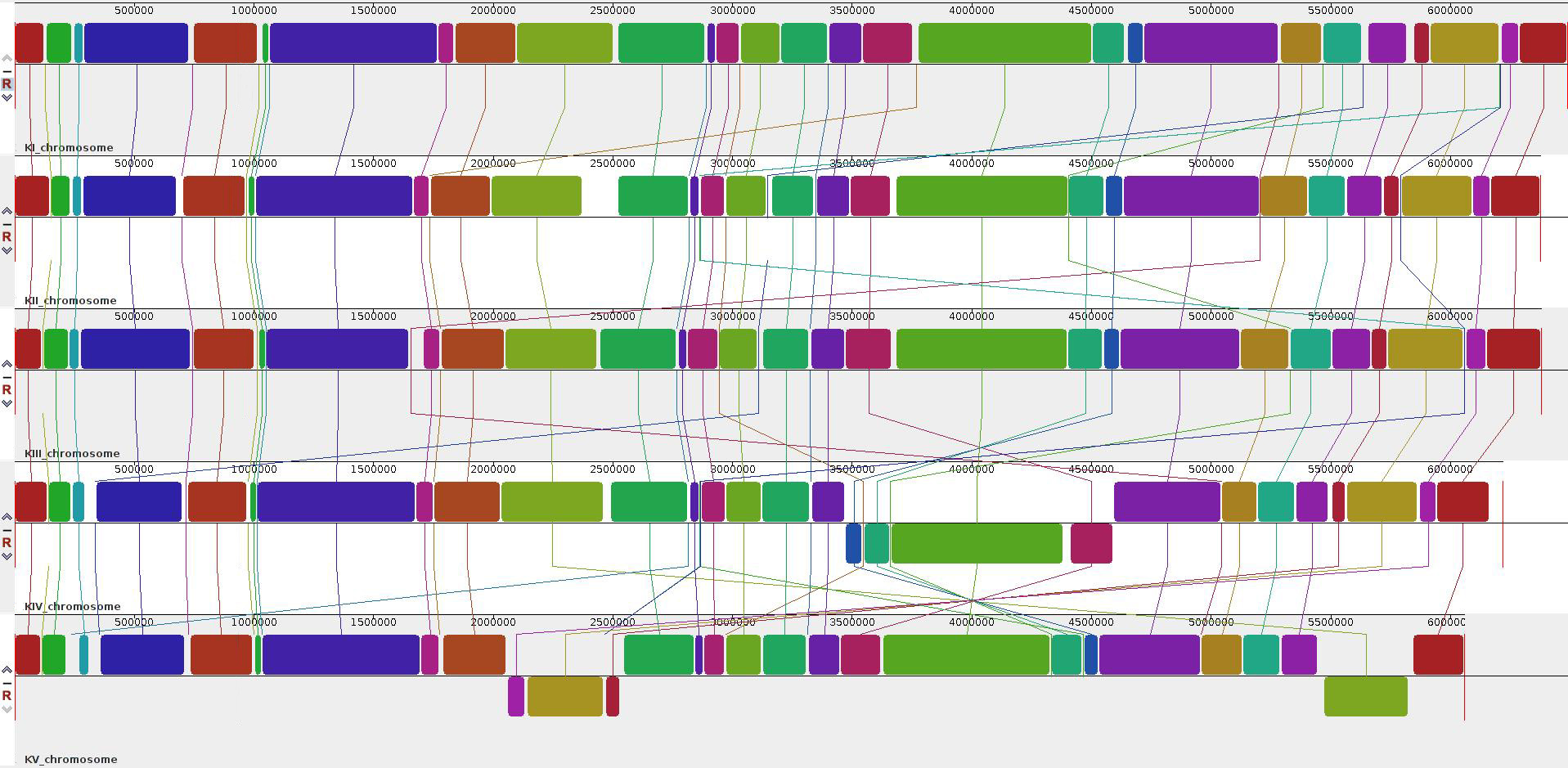
**

**Supplementary Figure 2**. Syntenic and conserved genome blocks of the five subtypes (strain KAUST-I~V) by aligning the assembled genomes with Mauve (Darling et al., 2004).


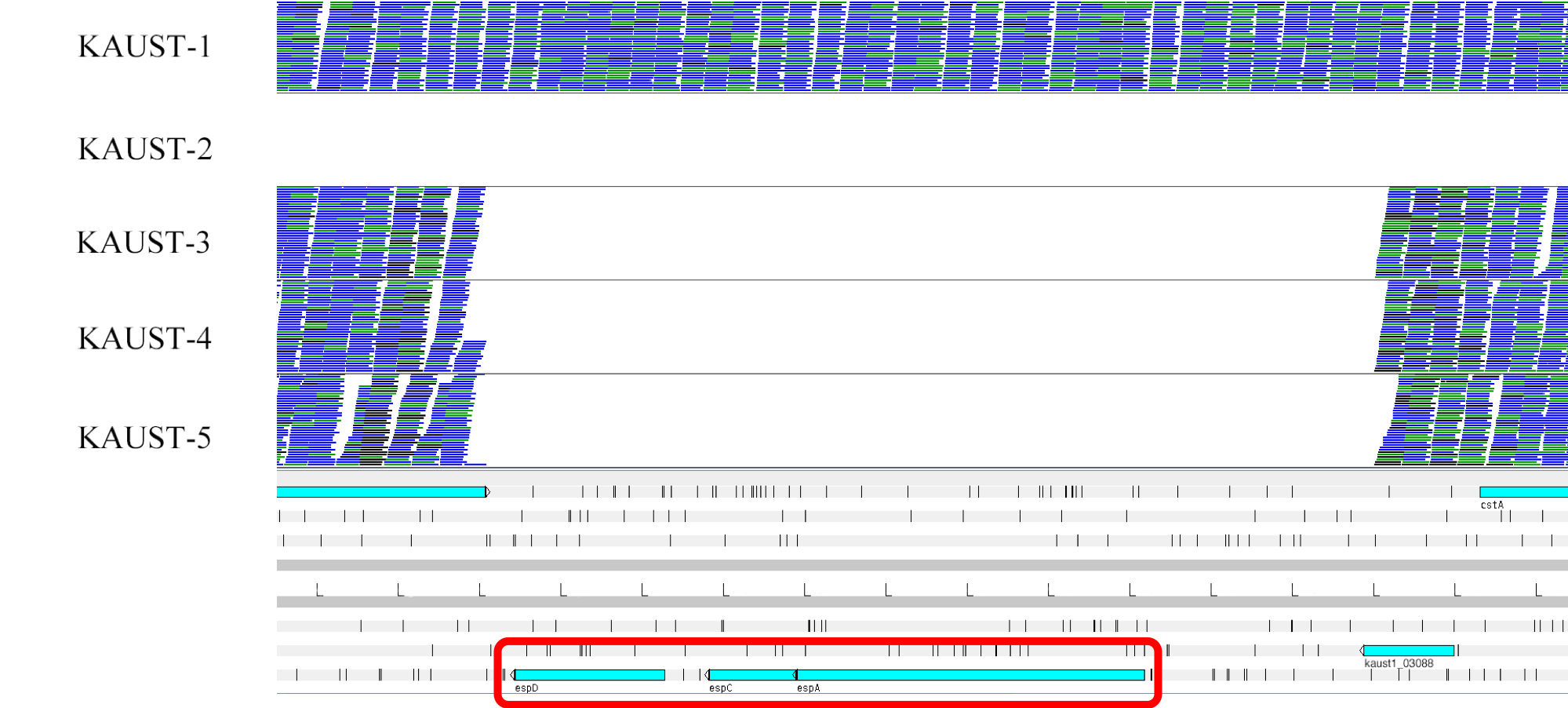


**Supplementary Figure 3.** The *espACD* operon region (subtype I) in *M. kansasii* strain KAUST-I. Displaying the presence of the *espACD* operon in *M. kansasii* subtype I while absent in the other four subtypes. BAM alignment files from each subtype mapping to the *espACD* loci in subtype I was viewed in Artemis (Rutherford et al., 2000) for the illustration.


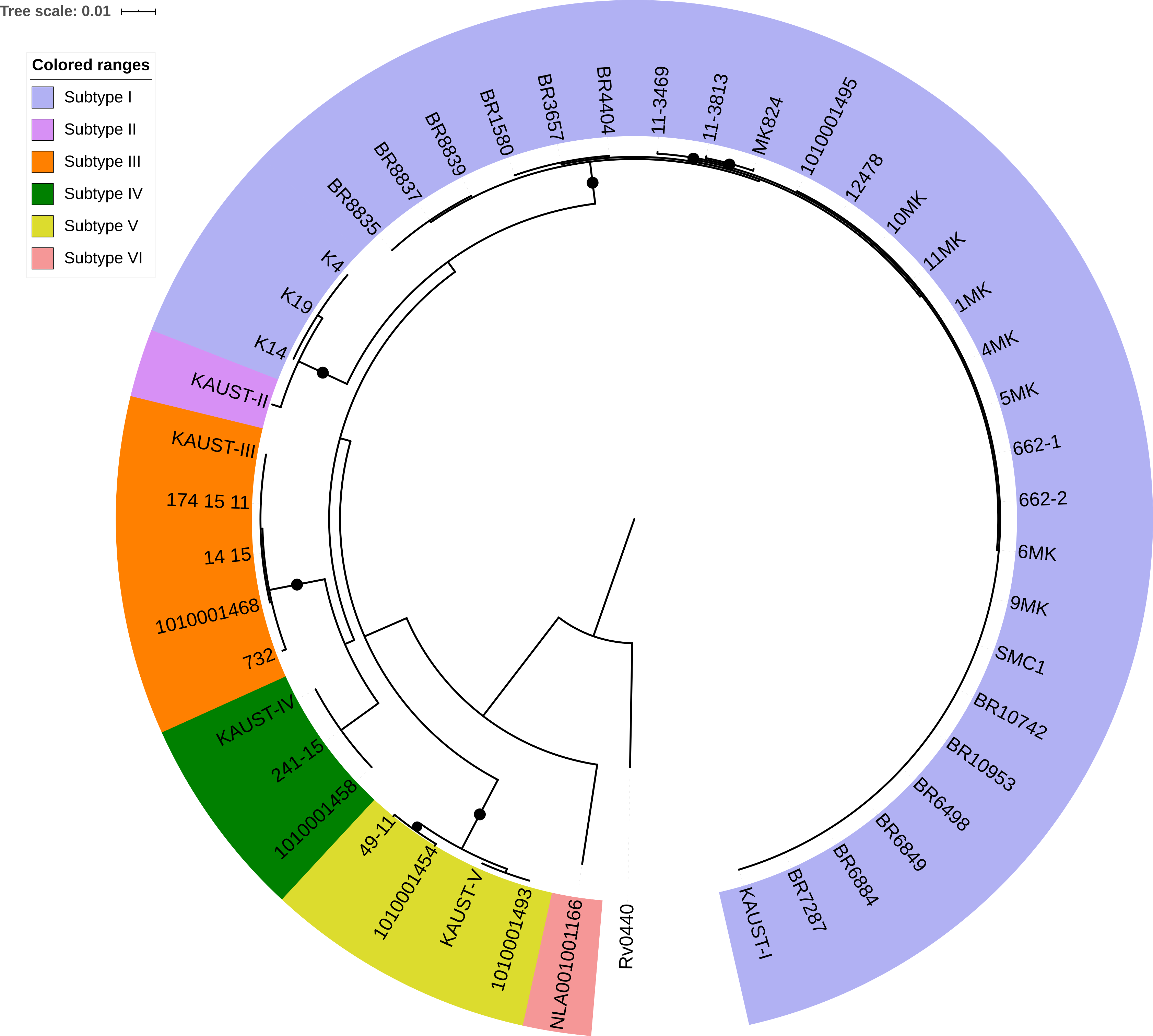


**Supplementary Figure 4.** Maximum-likelihood *hsp65* gene-based phylogenetic tree of *M. kansasii* genotypes across the forty-five sequenced *M. kansasii* strains. RaxML (Stamatakis, 2014) maximum-likelihood method (Stamatakis, 2014) with 100, 000 replicates was used to generate this phylogeny. Bootstrap values above 80% are indicated as the blue circles on the branches.


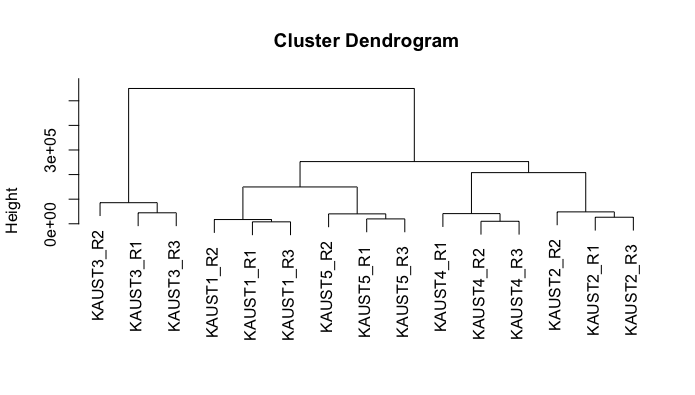


**Supplementary Figure 5.** Euclidean distance matrices of RNA-seq transcriptome HTseq (Anders et al., 2015) count matrix showing clustering of *M. kansasii* subtype I-V strains grown in culture medium (three biological replicates).

**
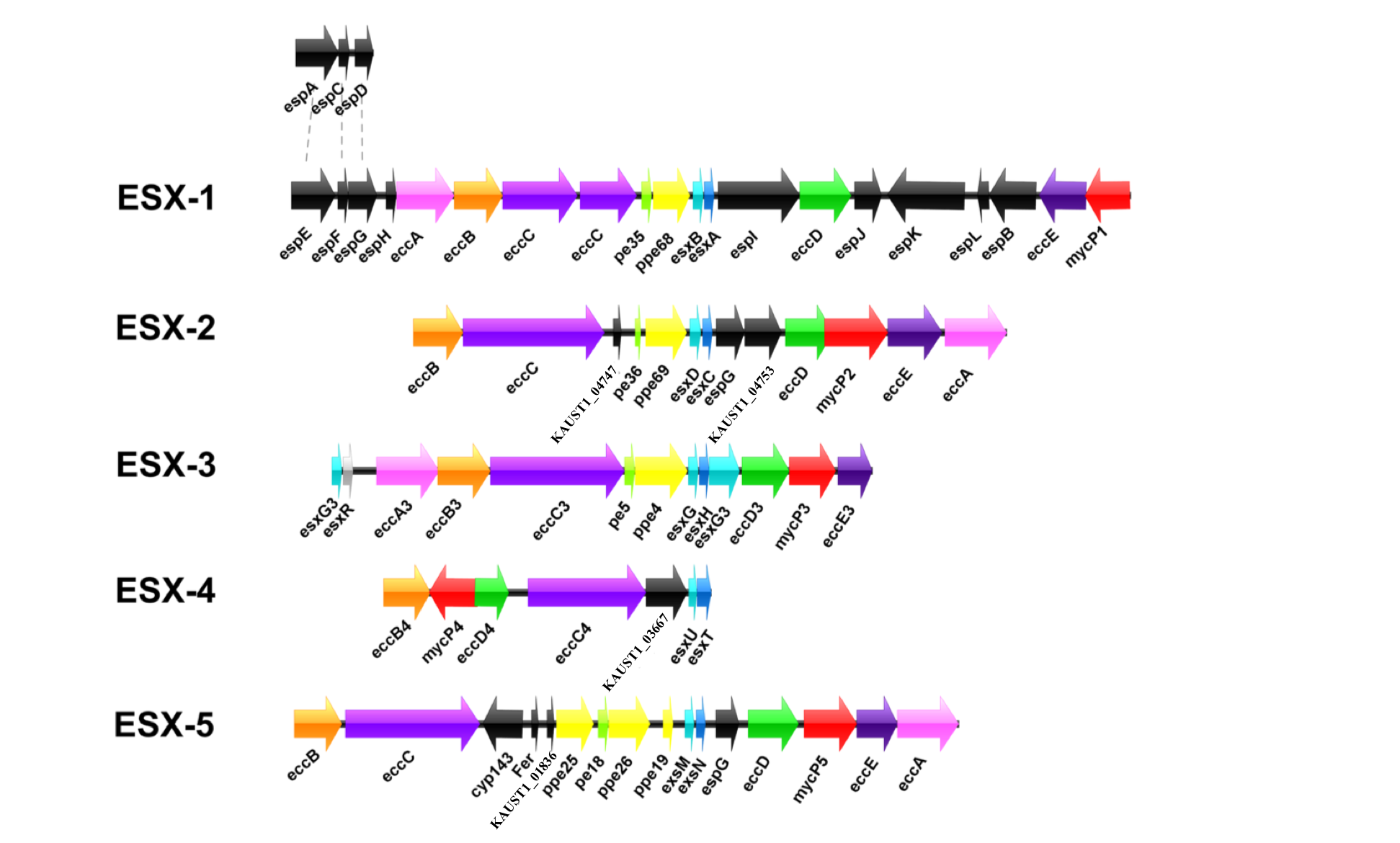
**

**Supplementary Figure 6.** A schematic representation of the ESX loci in *M. kansasii* subtype I (drawn to scale).


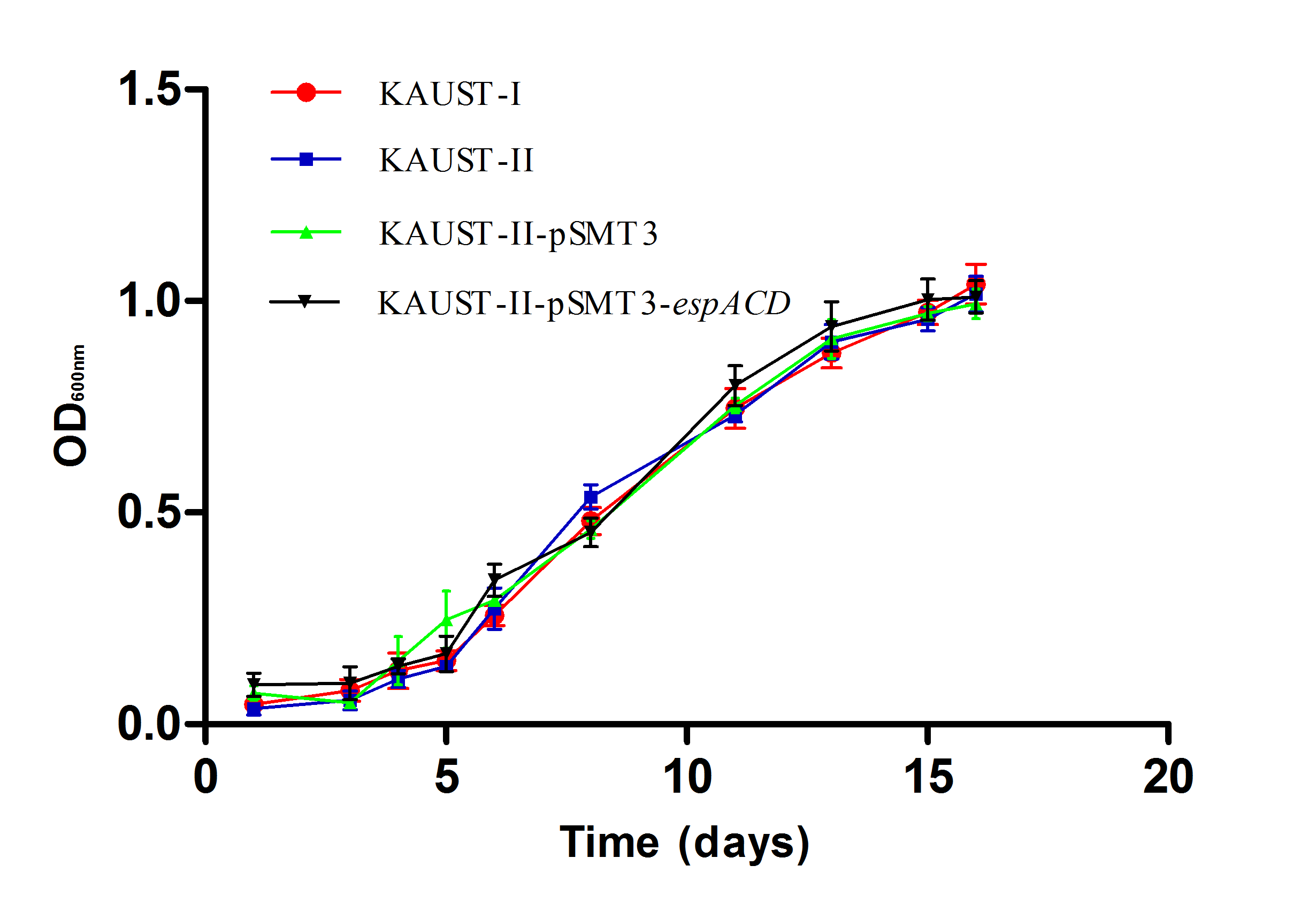


**Supplementary Figure 7**. Growth curve of different *M. kansasii* strains: *M. kansasii* KAUST-I, KAUST-II, KAUST-II-pSMT3, KAUST-II-pSMT3-*espACD*.

**Reference**

Anders, S., Pyl, P. T., and Huber, W. (2015). HTSeq-A Python framework to work with high-throughput sequencing data. *Bioinformatics* 31, 166–169. doi:10.1093/bioinformatics/btu638.

Darling, A. C. E., Mau, B., Blattner, F. R., and Perna, N. T. (2004). Mauve: Multiple alignment of conserved genomic sequence with rearrangements. *Genome Res.* 14, 1394–1403. doi:10.1101/gr.2289704.

Rutherford, K., Parkhill, J., Crook, J., Horsnell, T., Rice, P., Rajandream, M. a, et al. (2000). Artemis: sequence visualization and annotation. *Bioinformatics* 16, 944–945. doi:10.1093/bioinformatics/16.10.944.

Stamatakis, A. (2014). RAxML version 8: A tool for phylogenetic analysis and post-analysis of large phylogenies. *Bioinformatics* 30, 1312–1313. doi:10.1093/bioinformatics/btu033.
